# Concern on cyber violence and suicide during COVID-19 pandemic

**DOI:** 10.3389/fpsyt.2022.956328

**Published:** 2022-09-07

**Authors:** Zhiwei Liu, Rongchun Yang, Huanzhong Liu

**Affiliations:** ^1^Department of Psychiatry, The Third People's Hospital of Fuyang, Fuyang, China; ^2^Department of Psychiatry, Chaohu Hospital of Anhui Medical University, Hefei, China

**Keywords:** cyber violence, COVID-19, prevalence, pandemic, suicide

## Introduction

Three days after delivering food to her father, she jumped to her death. It started when a storm of public opinion erupted after she gave the delivery man $30.76 for his kindness. This real case occurred during the COVID-19 pandemic in Shanghai in April 2022. The girl, who had a happy family and was physically and mentally healthy, shared her heartwarming story on her Micro-blog, thanking the delivery man for his selfless act. However, this “small” incident cost her life.

Previous studies have shown a significantly rise in suicide rates during the COVID-19 pandemic (1, 2). In addition, current research indicates that cyber violence is closely associated with a higher risk of suicidal behavior (3, 4). Under the double pressure of epidemic and cyber violence, such tragic events are by no means negligible. Relevant departments should step up network security monitoring, timely block the injury of cyber violence to the victims, and provide psychological intervention for the victims of cyber violence, in order to prevent the occurrence of suicide.

Cyber violence is toxic to society. It refers to the illegal and criminal behavior by which individuals or groups intentionally spread illegal information through the Internet to repeatedly and continuously infringe on specific individuals or groups, which will lower the social evaluation of the attacked individuals, infringe on the right of personality and information, and even threaten the rights of person and property (5). The perpetrators of cyber violence have a degree of anonymity that traditional bullying does not, and the potential exposure and embarrassment for victims is greater. Day or night, in their own homes or anywhere else, harm can be done to victims, and the information accumulates even when they leave the Internet. Victims of cyber violence often have mental health problems, including symptoms of depression, anxiety, and self-harm, up to suicide (6). Previous study revealed that the prevalence of cyber violence ranged up from 6 to 35% before the epidemic (7). However, cyber violence has increased significantly during the pandemic (8).

## Shadows of the pandemic: Cyber violence and suicide

In the early days of the COVID-19 pandemic, The Lancet Psychiatry Commission predicted an increase in suicide rates worldwide (9). Significantly, a critical review went on to confirm this revelation that rates of suicidal ideation during the COVID-19 pandemic were higher than those reported in pre-pandemic studies of the general population (1, 2). Moreover, women need special support for higher risks of suicidal behavior during the pandemic, as they are at greater risk of unemployment, have heavier family burdens and face more violence from their families or society during lockdowns and crises (10).

The United Nations has identified violence against women (VAW) as a shadow pandemic created in the midst of the COVID-19 pandemic. Previous literature indicated that the incidence of cyber violence against female victims was significantly higher than that against males (11), and cyber violence was closely associated with increased suicidal ideation (12). Looking at cyber violence as a form of VAW during the pandemic, a recent study found that almost 25% of respondents frequently observed different forms of cyber violence against women and girls (13). While cyber violence against women significantly increased during the pandemic (14), there were few rigorous studies on the relationship between the COVID-19 pandemic and VAW. Data from those studies consisted mostly of articles published in comments, editorials, and letters from social media, the Internet, and helpline reports. Research on the COVID-19 pandemic and cyber violence is even rarer.

A review that included reports from 53 WHO European member states found that the most common measure to prevent or respond to VAW was to use social media to raise awareness of violence among female victims and to provide services to them through online platforms, followed by measures to expand or maintain helpline services for female victims of violence (15). Some people may not seek help because they fear that face-to-face appointments could put them at risk and emergency services are overwhelmed in the pandemic. Additionally, due to the anonymity of cyber violence and the complexity of obtaining evidence (16), it is quite difficult to establish the rights protections of victims themselves or the jurisdiction of law enforcement departments. Others may look for help from crisis helplines in the government sector, which are likely to be overwhelmed by a surge in calls and shortage of service personnel.

## Discussion

In this case, the suicide occurred just 2 days after the outbreak of cyber violence. It can be seen that the intervention of suicidal behavior lacks timeliness. During the COVID-19 pandemic, the incidence of cyber violence and suicide has increased, respectively (17, 18). It is hoped that concerted efforts will be made to prevent the occurrence of adverse events related to cyber violence. Unfortunately, little research has been done on the relationship between these two issues. There are several recommendations of our paper: Online entertainment websites and platforms, while seeking to maximize their products, should fulfill their moral responsibilities by controlling online content, formulating and publicizing internet anti-abuse policies, regularly testing their impact, and investigating whether users have recently suffered from cyber violence, so as to point out a direction for users' psychological support. For the general public, it is necessary to control unnecessary Internet use time, carry out more offline physical exercise, and maintain good peer and family relations (7). We should strictly take legal action or hold perpetrators accountable, provide psychological support to victims and witnesses in a timely manner, and try our best to ease their psychological trauma. All health professionals, especially those in mental health, should do their best to educate clients and families about steps to stay Internet-use safe and take timely intervention for the victims. Mental health services should develop clear pathways for remote assessment and care, and train staff in new ways of working during an epidemic to provide timely and effective interventions for people at risk of suicide. At the same time, at the national level, online resources and psychological interventions should be provided and promoted free of charge on a large scale for the basic mental health of the vast population (9). Health care systems should facilitate further investigation into the relationship between violence against women, especially cyber violence, and COVID-19 to identify creative solutions to deliver clinical care and medical services to the victims.

## Public health implications

The issue of cyber violence during the pandemic is extremely important and cannot be ignored. Relevant departments need to formulate rigorous and effective measures to reduce the suicide caused by it.

## Author contributions

HL originally designed the study and have been responsible for obtaining funding. All authors contributed to this study, read the manuscript, approved the final manuscript, interpretation of data, and the approval of the final report.

## Funding

This paper was supported by Anhui Institute of Translational Medicine Research Fund (No. 2017zhyx17) and the Anhui Provincial Key R&D Program (202004j07020030).

## Conflict of interest

The authors declare that the research was conducted in the absence of any commercial or financial relationships that could be construed as a potential conflict of interest.

## Publisher's note

All claims expressed in this article are solely those of the authors and do not necessarily represent those of their affiliated organizations, or those of the publisher, the editors and the reviewers. Any product that may be evaluated in this article, or claim that may be made by its manufacturer, is not guaranteed or endorsed by the publisher.
